# Adulteration of Rape-Cake with Mustard

**Published:** 1855

**Authors:** 


### ADULTERATION OF RAPE-CAKE WITH MUSTARD.

Professor Voelcker has furnished to the *Agricultural Gazette* of January 20th some valuable information on the adulteration of rape-cake with mustard-seed. The food thus adulterated has, in several instances, proved poisonous to cattle. Dr. Voelcker has made some chemical examinations of the substance in question. "Apparently it is a sweet-smelling and good-looking rape-cake, from which, indeed, it cannot easily be distinguished on simple inspection. It is, however, a cake which is sure to kill any animal, if used for feeding purposes. This cake, I have found on examination, consists entirely of the expressed seeds of mustard. My attention was directed to the subject by Mr. —, who had two beasts killed in consequence of their having eaten a small quantity of the cake. Having tested the cake in vain for arsenic, lead, copper, and other metallic poisons, I found that some of it, digested in cold water, in the course of a couple of hours, had become as pungent as *mustard;* and, on distillation, I obtained in reality the highly irritating, characteristic smelling, volatile oil of mustard." Cases of poisoning of cattle with mustard are not unfrequent. The detection of the fraud is not difficult, if it is remembered that the volatile oil of mustard is only developed very gradually if the powdered seed is moistened with cold or lukewarm water. It does not exist in the whole seed; and its production is prevented by boiling water.

